# Ligand-Based Stability Changes in Duplex DNA Measured with a Microscale Electrochemical Platform

**DOI:** 10.3390/bios9020054

**Published:** 2019-04-12

**Authors:** Sarah M. Robinson, Zuliang Shen, Jon R. Askim, Christopher B. Montgomery, Herman O. Sintim, Steve Semancik

**Affiliations:** 1 Department of Chemistry and Biochemistry, University of Maryland, College Park, MD 20742, USA; smenio@umd.edu (S.M.R.); zuliangs@gmail.com (Z.S.); 2 Department of Chemistry, Institute for Drug Discovery, Purdue University, West Lafayette, IN 47907, USA; 3 Biomolecular Measurement Division, National Institute of Standards and Technology, Gaithersburg, MD 20899, USA; jon.r.askim@gmail.com (J.R.A.); chip@nist.gov (C.B.M.)

**Keywords:** DNA, electrochemical sensor, ligand-based stabilization, melting profiles, microfabrication, microheater, rapid temperature control, square wave voltammetry

## Abstract

Development of technologies for rapid screening of DNA secondary structure thermal stability and the effects on stability for binding of small molecule drugs is important to the drug discovery process. In this report, we describe the capabilities of an electrochemical, microdevice-based approach for determining the melting temperatures (*T_m_*) of electrode-bound duplex DNA structures. We also highlight new features of the technology that are compatible with array development and adaptation for high-throughput screening. As a foundational study to exhibit device performance and capabilities, melting-curve analyses were performed on 12-mer DNA duplexes in the presence/absence of two binding ligands: diminazene aceturate (DMZ) and proflavine. By measuring electrochemical current as a function of temperature, our measurement platform has the ability to determine the effect of binding ligands on *T_m_* values with high signal-to-noise ratios and good reproducibility. We also demonstrate that heating our three-electrode cell with either an embedded microheater or a thermoelectric module produces similar results. The Δ*T_m_* values we report show the stabilizing ability of DMZ and proflavine when bound to duplex DNA structures. These initial proof-of-concept studies highlight the operating characteristics of the microdevice platform and the potential for future application toward other immobilized samples.

## 1. Introduction

Many small molecules (i.e., ligands) that bind to DNA have been investigated as anti-cancer therapeutics [1,2,3]. Starting with the discovery of the changes in bone marrow caused by mustard gas poisoning in as early as 1917, advances related to ligand binding to DNA have continued over the last century [4]. Molecules that interact with duplex DNA breaks while bound to topoisomerases, critical to DNA transcription and replication, can cause replication miscoding or prevent the proper processing of supercoils in DNA, and therefore can be used as anticancer or antibacterial drugs [5]. Duplex DNA binders, such as doxorubicin and daunorubicin, have already yielded clinical successes for the treatment of cancers such as breast cancer, esophagus cancer, liver cancer, osteosarcomas, non-Hodgkin’s lymphoma, and acute myeloid leukemia [6,7,8]. On the other hand, DNA binding compounds cause mutagenicity, so, if duplex DNA is not the intended target, binding could cause undesired toxicity effects [9].

Microscale platforms such as the one presented here could enable the rapid screening of ligands with respect to their binding properties to duplex or other DNA structures, and thus facilitate the drug discovery process. Nucleic acid binders have previously been identified via melting-curve analysis of nucleic acids in the presence/absence of ligands [10,11,12]. When screening ligands as potential drug molecules, those that bind to and stabilize the duplex, causing shifts in the melting temperature, *T_m_*, can be considered “hits”. If duplex DNA is the intended drug target, these “hits” indicate compounds that warrant further investigations including structure–activity relationship (SAR) studies. If duplex DNA is not the intended drug target, these “hits” could be used when screening a library of compounds with other known targets for potential genotoxicity. 

The majority of studies that use melting-curve analysis have employed optical monitoring systems [12,13]. A complementary approach, amenable to development of electronic array formats and hence high-throughput screening, is melting-curve monitoring via electrochemical detection of electrode-tethered DNA. Herne and Tarlov pioneered DNA assembly and characterization of probe sequences tethered with thiol linkers [14,15]. In the early 2000s, Plaxco and co-workers developed these technologies further, outlining a protocol that uses methylene blue (MB)-labeled DNA for electrochemical DNA (E-DNA) sensors [16]. Over the past few decades, research has expanded in a variety of applications using thiol-modified DNA on gold surfaces [17,18,19,20].

Temperature control is a critical variable for investigating kinetics and thermodynamics of biomolecular materials including DNA. Having a heat source that is compatible with microdevice architectures opens the opportunity of arrays with locally heated sites. Prior examples of coupling electrochemical detection with controlled temperature capability include: thermoelectric modules [21,22], heating wells of solutions [23,24] and heated wire electrodes [25,26]. The incorporation of copper resistive heaters on the backside of fiberglass epoxy sheets has been used for quantitative polymerase chain reaction (PCR) biosensors [27]. Fiche et. al. used a heated flow cell to analyze the thermal stability of DNA with surface plasmon resonance (SPR) [28], and Buhot continued the approach to work with Au nanoparticles (AuNP) for SPR imaging [29]. Additional work was done by Bartlett and co-workers on denaturing DNA by electrostatic repulsion and monitoring the process through surface-enhanced Raman spectroscopy (SERS) [30,31]. The Levicky group used disk electrodes to analyze the thermal stability of DNA–ligand complexes, and the sample volumes in such studies are governed by the size of the electrodes [23]. Additionally, the Plaxco and Soh groups created a microelectrochemical dynamic allele-specific hybridization device (MicroE-DASH) [32]. The results of these studies and other prior work have shown that immobilization of DNA to the surface affects enthalpies and entropies of hybridization when compared to standard solution-phase methods [23,33,34].

Previous studies by our group demonstrate the utility of microheater systems integrated with a three-electrode electrochemical cell to detect single or multiple base mismatches in immobilized duplex DNA [35] and to study temperature-induced enhancement of signals due to temperature-dependent diffusion and thermal convection in model redox systems [36]. Our platform is unique in having an individual embedded platinum heater integrated with each planar three-electrode set, thus positioning our methodology for easy scale-up to addressable arrays such as those for studying immobilized DNA under a variety of conditions. The microheater provides programmable, localized heat and also functions as a thin-film platinum resistance thermometer (PRT), probing resistance and thus temperature less than 1 µm from the electrodes. The platinum heater is insulated from the surface three-electrode system by an SiO_2_ layer varying in thickness from 350 nm to 1 µm depending on device generation. To extend the temperature range for analysis below room temperature, the electrochemical platform can be contained in a refrigerated box, or an external thermoelectric (TE) module can be placed on the backside of the wafer for localized cooling. When the thermoelectric module is used as both a cooling and heating source, the embedded platinum heater is still employed as a PRT to monitor temperature. A polydimethylsiloxane (PDMS) sample microchamber mounted above the electrodes contains the analyte solution. This configuration enables rapid temperature ramping and independent current monitoring.

**Figure 1:**
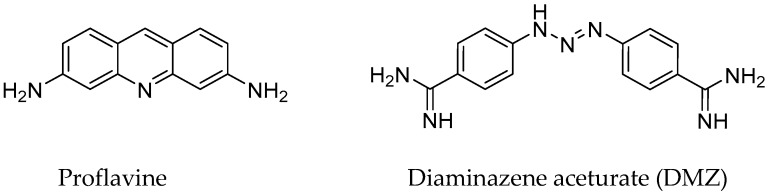
Structures of ligands used in this study.

Herein, we demonstrate the use of our temperature-controlled electrochemical microplatform in two different assemblies, namely with a resistive heater, or with a thermoelectric module, to perform duplex DNA melting in the presence/absence of proflavine, a well-known intercalator and one of the first published DNA binders [37,38], and diminazene aceturate (DMZ or Berenil), a minor groove binder [39,40]. Chemical structures of the examined binders are shown in Figure 1. For investigating DNA, a full-matched duplex was formed by an immobilized 5’-thiolated, single-stranded DNA (ssDNA) hybridized with a complementary sequence labeled with a redox-active MB on C7 of the 3′ end. The results of our previous study, which involved investigating single-nucleotide polymorphism (SNP) in immobilized DNA [35], motivated us to further explore whether changes in DNA melting temperature, *T_m_*, in the presence of DNA ligand binders could also be assessed using our platform. While our proof-of-concept work was done with known duplex DNA binders, the method could be easily employed to test induced stability changes for a variety of other ligands. We note that these selected ligands have also shown binding to other nucleotide structures such as c-di-GMP and G-quadruplexes [40,41,42]. The magnitude of changes in *T_m_* upon binding of these ligands was readily observable. In this paper, we additionally discuss specific enhancements to the performance of the microheater and the SiO_2_ insulating layer for increased robustness as well as the incorporation of measurement automation routines to decrease measurement variability.

## 2. Materials and Methods

### 2.1. Design of Microscale Platform

**Figure 2:**
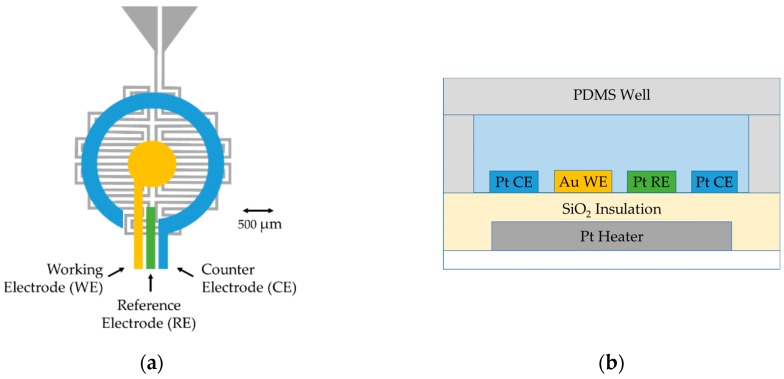
The electrochemical microscale platform architecture: (**a**) a top-view schematic of the platform (without the PDMS sample chamber); and (**b**) a side-view schematic (not to scale), including a sample well. The Pt heater/PRT fabricated on a glass wafer was isolated by a SiO_2_ insulating layer. Au and Pt electrodes were fabricated by two lift-off e-beam evaporated depositions. The PDMS chamber and lid were centered above the electrode system.

The microscale platform shown schematically in Figure 2 was fabricated on a fused silica wafer, a material with low thermal conductance, which allows for rapid, localized temperature ramping and promotes a stable heating profile at the electrochemical interface. A DC current was applied to the platinum embedded heater and the nearby area was heated via resistive heating. The ~650 Ω heater was designed in a bifilar, serpentine pattern to provide more uniform heating while minimizing any field effects through antiparallel current flow. The resistance of the platinum heater was approximately linear with a thermal coefficient of resistance large enough to make a practical thermometer. After calibration, resistance measurements of the heater provided direct, local measurement of the device temperature in real time. The platinum heater was isolated from the three planar top-surface electrodes with a layer of silicon oxide, which provides dense and pinhole-free insulation. The electrode system deposited above the insulation layer allowed electrochemical current measurements at different temperatures. Analyte sample solution was contained over the electrode area in a PDMS chamber with a PDMS lid to minimize evaporation, and the entire chamber is removable for piranha cleaning of the electrodes. The small footprint and thin profile of the microscale system led to an overall low thermal mass that allowed effective heat transfer for small-volume sample measurement (generally ≤10 µL). All sensor components were integrated for electrochemical measurements within the microchamber and can be readily adapted to develop multi-element array devices for high-throughput screening.

### 2.2. Materials

The oligomers for the duplex DNA formed by ssDNA (5′-S-S-(C_6_H_12_)-TTT ACC TTT ATT-3′) and cDNA (3′-(MB)-AAA TGG AAA TAA CC-5′) were synthesized by and purchased from Integrated DNA Technologies* (Coralville, IA, USA) and Biosearch Technologies* (Novato, CA, USA), respectively. Dimethyl sulfoxide (DMSO), diminazene aceturate (DMZ), 6-mercaptohexanol, proflavine hemisulfate salt hydrate, sodium phosphate dibasic, sodium phosphate monobasic, magnesium chloride heptahydrate, sodium chloride, and tris-(2-carboxyethyl) phosphine hydrochloride (TCEP) were purchased from Sigma-Aldrich* (St. Louis, MO, USA). All chemical reagents were of analytical grade or higher. Stock solutions of proflavine and DMZ were prepared at 14 mmol/L in DMSO and then diluted to 140 µmol/L in PBS buffer (10 mmol/L phosphate-buffered saline, pH 7.4) with 100 mmol/L NaCl. Deionized ultra-filtered (DIUF) water (18.2 MΩ·cm) was used in all preparations. Fused silica wafers were purchased from University Wafers* (Boston, MA, USA). 

### 2.3. Microfabrication of Three-Electrode Device with Embedded Microheater

**Figure 3:**
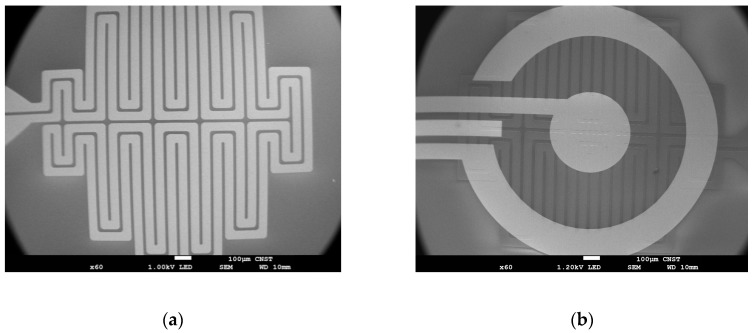
Scanning electron micrograph (SEM) of the embedded microheater: before electrodes were patterned on top (**a**); and after the fabrication was complete (**b**).

Fabrication was done using the National Institute of Standards and Technology (NIST) Center for Nanoscale Science & Technology (CNST) cleanroom facilities. To increase the lifetime of the devices relative to devices that we previously reported [35], fabrication steps were modified to increase both the quality of the platinum heater and the electrical and defect properties of the silicon oxide insulator. The new fabrication design uses an ion beam sputtering cluster tool (4Wave Inc.*, Sterling, VA, USA) with two process modules, biased target deposition and ion beam deposition, both including an etch gun for in situ precleaning. The wafers were etched for 60 s (at 20.6 nm/min) to expose fresh SiO_2_ and coated with a Ti adhesion layer (5 nm at 0.86 nm/min). Then, a Pt layer was deposited (220 nm, 2.35 nm/min) and capped with an additional Ti adhesion layer (5 nm at 0.86 nm/min). The wafer was transferred to the other process module, and either a SiO_2_ for the resistive heated wafer (50 nm, 2.31 nm/min) or an Al_2_O_3_ seed layer for the thermoelectric heated wafer (50 nm, 5.24 nm/min) was deposited in another ion beam deposition process module without breaking vacuum. Afterwards, lithography was used to pattern the heater mask for the ion beam milling (4Wave Ion Mill, 4Wave Inc.*), thereby creating the etched heater. The photoresist was removed using Microposit Remover 1165 (Dow Chemical Company*, Marlborough, MA, USA) and rinsed with acetone, isopropyl alcohol, and then DIUF water. The clean wafers were etched for 7–9 min at low power in the ion mill to round the edges of the heater traces. For the wafer used for the thermoelectric heating, the SiO_2_ layer was deposited (75 nm, 0.15 nm/cycle, 500 cycles) with remote plasma atomic layer deposition (ALD) (Oxford* FlexAL atomic layer deposition, Bristol, UK), then a second layer (200 nm, 112.3 nm/min) was deposited with plasma-enhanced chemical vapor deposition (Plasma-Therm* Versaline High Density Plasma Chemical Vapor Deposition (HDPCVD), Saint Petersburg, FL, USA), and lastly it was capped with an additional ALD SiO_2_ layer (75 nm, 0.15 nm/cycle, 500 cycles). For the wafer used with the embedded heater, 1 µm of HDPCVD oxide was deposited on top of the etched heater traces. The wafer was then annealed in a nitrogen furnace at 800 °C for 1 h (Sandvik* MRL Diffusion Furnace, Sandviken, Sweden). The top electrodes were patterned with lithography, and an e-beam evaporator (Denton* Infinity 22 E-Beam, Morristown, NJ, USA) was used to deposit a Ti adhesion layer (20 nm, 6.0 nm/min), and then the Pt reference and counter electrodes (200 nm, 6 nm/min). The process was repeated for the gold working electrode (200 nm, 6.0 nm/min), which has an exposed area of approximately 0.003 cm^2^. Once the fabrication was complete, the contact pads were patterned with lithography, and the SiO_2_ was etched with a reactive ion etcher (Plasma-Therm*/Unaxis* 790 RIE, Saint Petersburg, FL, USA). Figure 3 shows the top-view of the fabricated serpentine heater (Figure 3a) and a completed device (Figure 3b). 

### 2.4. PRT Calibration

The electrical resistance of platinum has a linear dependence on temperature (≈1 °C/Ω) in the temperature range of our experiments. With both assemblies described in Section 2.5 and Section 2.6, the embedded platinum thin-film was used also as a platinum resistance thermometer (PRT). Each PRT was calibrated independently due to the small center-to-edge difference in uniformity of the platinum deposition, which caused slight variations in the resistance values from device to device. For calibration, the device was placed in a convection oven (Cole-Parmer* Stable Temp 52412-78, Vernon Hills, IL, USA), and the temperature was increased in 10 °C increments and allowed 20 min to equilibrate at each temperature point. Two type K thermocouples (Omega* HH501BK) were used to probe the temperature inside the oven and obtain an average temperature at each set point. The associated Pt resistance vs. temperature was determined from current measurements from a source measure unit (SMU) (B2902A Keysight Technologies*, Santa Rosa, CA, USA) at 0.5 V. The resulting linear least squares fit of the calculated resistance vs. measured temperature was used to report temperatures from resistance measurements made during the melting experiments (Figure S1).

### 2.5. Platform Assembly: Resistive Heating

A 6 mm hole was milled through the custom printed circuit board (PCB) centered under the device mounting location to reduce thermal anchoring by providing an air gap on the back of the device and to also allow contact of a thermoelectric device to the backside of the wafer (described in Section 2.6). Completed wafers with 1 µm PECVD SiO_2_ were diced into individual devices and mounted on the PCB with epoxy. The leads of the device were wire bonded to PCB traces for easy electrical connection. The mounted device was placed in a polystyrene foam box with an ice pack and allowed to equilibrate for 30 min before analysis. A SMU supplied a voltage for resistive heating and also monitored the current through the Pt heater (thereby permitting temperature monitoring). A voltage of 0.5 V was used to monitor the temperature during the equilibration time and then the voltage was increased by 0.4 V every 25 s from 1.0 V to 11.4 V to produce the stepped temperature ramp for the melting experiments. 

### 2.6. Platform Assembly: Thermoelectric Heating

Devices fabricated with 350 nm SiO_2_ were used for the thermoelectric heating studies with the Pt thin-film used as the PRT for temperature monitoring. As described in Section 2.5, a 6 mm hole was milled through the PCB and the completed device was secured to the board with epoxy so that the hole in the PCB was centered under the PRT. Room-temperature-vulcanizing (RTV) silicone pillars were placed on an aluminum block heat sink with two screws on either side of the PCB for securing the back side of the glass wafer at the height of the thermoelectric module (Peltier MS2,010,06,06,11,11,00,W2, Laird Technologies Inc.*, Chesterfield, MO). A picture of the assembled platform can be found in Figure S2. The thermoelectric module was placed on an aluminum block and the PCB was screwed in place on top to ensure efficient heat transfer between the thermoelectric module, the backside of the wafer, and the aluminum heat sink. Dow Corning 340 (Dow Chemical Company*, Midland, MI, USA), a silicone heat sink compound, was used at the interface between the fused silica wafer and the top of the thermoelectric. The SMU supplied a voltage for thermoelectric heating or cooling of the thermoelectric module, and also monitored the current of the PRT. The sample was allowed to equilibrate at room temperature for 15 min; during that time, 0.001 V was applied to the thermoelectric module and 0.5 V to the PRT, allowing for temperature monitoring. To reach 10 °C, 0.2 V was applied for 20 min, and then for the heating cycle, the voltage was stepped by 0.25 V every 25 s from 0.2 V to −0.4 V. The temperature was monitored at 0.5 V while the sample was cooled and then heated, and this monitoring was terminated before each measurement of the electrochemistry program, to minimize any leakage current. The temperature value for each square wave voltammogram (SWV) was the last read out before electrochemistry program started. 

### 2.7. Electrode Cleaning and Preparation of Duplex Self-Assembly on Au Electrode

The Au working electrode was first cleaned with a drop of piranha solution (3:1 volume mixture of concentrated H_2_SO_4_ and 30% (w/w) H_2_O_2_ in H_2_O) for 2 min (Caution! Piranha solutions can be explosive if they contact organic materials), rinsed with DIUF water, and then electrochemically cleaned with 20 µL of 0.5 mol/L H_2_SO_4_. The potential was swept from 0 V to 0.9 V (vs. the platinum reference electrode) at a scan rate of 0.1 V/s for a total of twenty cycles. To reduce disulfide bonds in the ssDNA, 1 µL of 200 µmol/L ssDNA was mixed with 2 µL of 20 mmol/L TCEP in DIUF water at room temperature in the dark for 90 min. The solution was then diluted to a DNA concentration of 2 µmol/L with 100 µL PBS buffer (10 mmol/L phosphate-buffered saline, pH 7.4) with 1 mol/L NaCl and 1 mmol/L MgCl_2_. After cleaning, the Au electrode was incubated with 10 µL of 2 µmol/L ssDNA for 90 min in a dark, high-humidity chamber. The electrodes were thoroughly rinsed with DIUF water and dried with nitrogen. To form a self-assembled monolayer (SAM) and minimize nonspecific adsorption to the Au surface, the electrodes were incubated with 10 µL of 2 mmol/L 6-mercaptohexanol solution in PBS with 1 mol/L NaCl and 1 mmol/L MgCl_2_ for 1 h at room temperature, in a dark, high-humidity chamber. The electrodes were rinsed for 1 min using DIUF water to remove any remaining 6-mercaptohexanol solution, and again dried with nitrogen. To study the hybridization interactions, the PDMS chamber with a 2.5 mm diameter hole was filled with 10 µL of 2 µmol/L cDNA in PBS buffer containing 100 mmol/L NaCl; this sample was left to stand for 15 min at room temperature, and then cooled to 10 °C. The melting profile was performed as described in Section 2.5 or Section 2.6, and then samples were allowed to cool to room temperature over 15 min. When examining ligand stabilization, 1 µL of 140 µmol/L ligand stock solution was added to the PDMS well after the ~15 min at room temperature. The sample was then cooled to 10 °C for 20 min and the melting profile was analyzed again. After completion, the PDMS well was removed and the device was rinsed in DIUF water. Due to the possibility of microelectronic component degradation in buffer, the devices were stored dry and prepared fresh for each set of experiments.

### 2.8. Melting-Curve Analysis

For duplex DNA melting-curve analysis, all electrochemical measurements were performed with an electrochemical workstation (CHI1040c, CH Instrument Inc.*, Austin, TX, USA). Sample temperature was controlled and monitored (as discussed above for the two different set-ups in Section 2.5 and Section 2.6) using the embedded platinum thin film as a PRT in both cases.

The temperature was increased incrementally with selected voltage steps under computer control, 15 s were allowed to equilibrate the sample, and then 10 s were allowed to acquire the square wave voltammetry (SWV) scan for that temperature. The thermal profile of the temperature measurements is shown in Figure S3. SWV was carried out in all studies from −0.75 V to −0.35 V, or −0.70 to −0.30 V, with a 0.001 V interval, 60 Hz frequency and 0.025 V amplitude. Baseline correction was performed by fitting a straight line to the two lowest points on either side of the MB peak and then subtracting the baseline from the peak current to measure the peak height. All data were plotted in Origin (OriginPro 2016, OriginLab Corp*, Northampton, MA, USA) and a Boltzmann function was fit to the data to find the *T_m_*. In cases where the current had an initial increase at low temperature, at least one data point before the maximum was included in the Boltzmann function to find the T*_m_*. These values were also confirmed by taking the derivative of the thermal profile.

## 3. Results and Discussion

**Figure 4:**
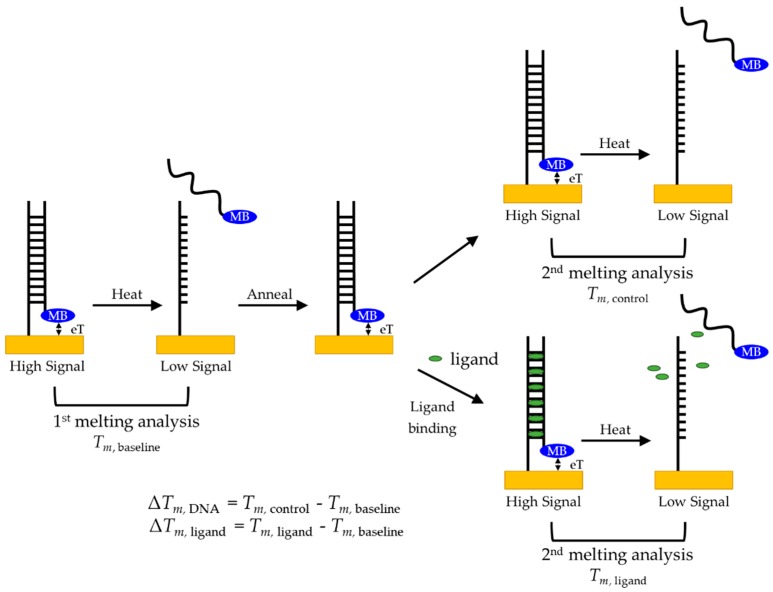
Melting analysis protocol for duplex DNA to probe for ligand-induced stabilization and to minimize measurement variability.

Because MB has a known reversible oxidation-reduction potential [16], we can monitor the current produced by the MB as a function of temperature. When the maximum amount of the complementary sequence is bound, the high relative concentration of MB proximal to the surface (see Figure 4) leads to high electron transfer (eT), and therefore produces the highest current. As the temperature increases incrementally, the equilibrium of bound and unbound complementary DNA shifts as less of the MB-labeled complementary sequence is hybridized, meaning the concentration of MB close to the surface decreases, causing the current measured to decrease. A melting curve was constructed by plotting the peak currents of MB at each temperature step vs. temperature, with the inflection point determining the melting point, *T_m_*. An increase in stability, such as that caused by ligands bound to the DNA probe, requires more energy to dehybridize the duplex, causing an increase of *T_m_* and thus a positive change, Δ*T_m_*. In addition, the more a ligand stabilizes the structure, the greater is the Δ*T_m_*.

To maximize the measurement repeatability for quantifying the effect introduced by a given ligand under specific conditions, we used a sequential melting protocol, illustrated schematically in Figure 4. Because we were interested in the shift, or Δ*T_m_*, a baseline measurement of the duplex DNA melting, *T_m_*_, baseline_, was taken before adding the ligand. After allowing the duplex to rehybridize, the sample was melted again either in the absence or in the presence of the ligand of interest. With this protocol, we could compare the Δ*T_m_* of no ligand added, Δ*T_m,_*_DNA_, to confirm that the hybridization is reversible. We then examined incubation with two molecules known to bind to duplex DNA, proflavine and DMZ, to determine whether we could quantify any change in stability caused by ligand binding, Δ*T_m,_*_ligand_. 

**Figure 5:**
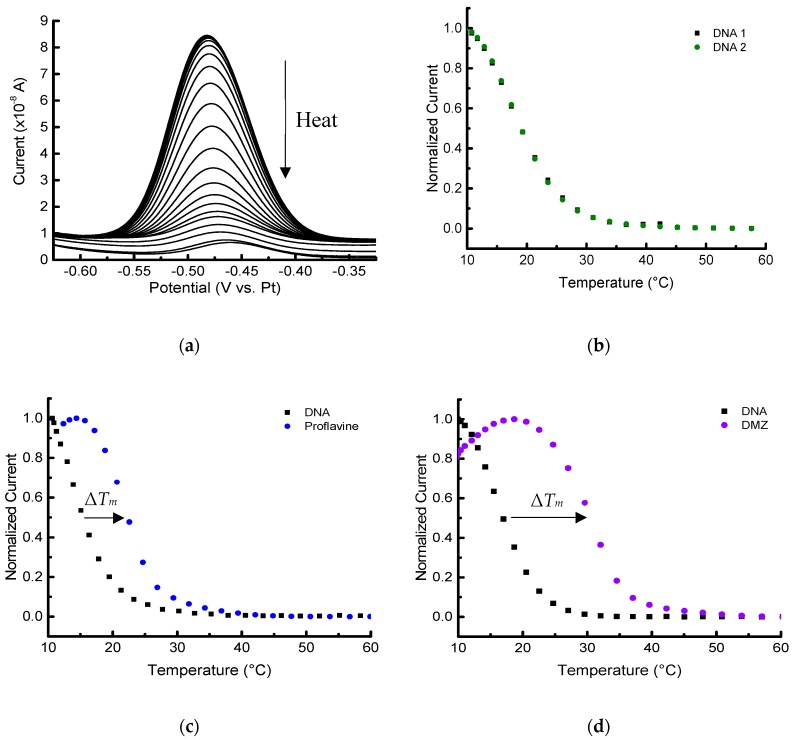
Melting-curve analysis of duplex DNA in the presence/absence of binding ligands performed with an embedded heater. (**a**) Square wave voltammograms of 2 µmol/L MB-labeled duplex DNA in 10 mmol/L PBS with 100 mmol/L NaCl, pH 7.4. Melting curves (normalized) of duplex in the absence (**b**) and presence of 13 µmol/L ligands: proflavine (**c**), and diminazene aceturate (DMZ) (**d**). (**b**–**d**) The first baseline curve is shown in black, and the second melting analysis with or without ligand is shown in its respective color.

**Figure 6:**
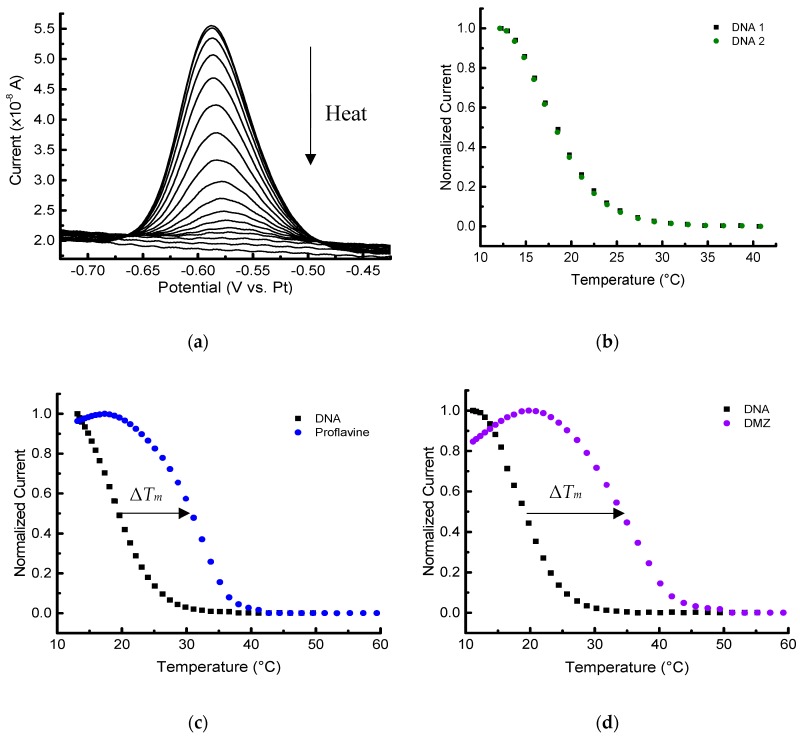
Melting-curve analysis of duplex DNA in the presence/absence of binding ligands performed with a thermoelectric module. (**a**) Square wave voltammograms of 2 µmol/L MB-labeled DNA duplex in 10 mmol/L PBS with 100 mmol/L NaCl, pH 7.4. Melting curves (normalized) of duplex in the absence (**b**) and presence of 13 µmol/L ligands: proflavine (**c**), and diminazene aceturate (DMZ) (**d**). (**b**–**d**) The first baseline curve is shown in black, and the second melting analysis with or without ligand is shown in its respective color.

Experiments to obtain melting curves for different samples were performed with two different methods for cooling and heating: (1) using an insulated container for cooling and the embedded platinum thin film for resistive heating (Figure 5); and (2) using a thermoelectric (Peltier) module to cool and heat the device while monitoring the temperature with the PRT (Figure 6). Each of these methods has its own advantages. For example, a resistive heating device is relatively inexpensive compared to a Peltier module for temperature control, and it offers a straightforward approach to run many devices in parallel with different thermal profiles.

The results shown in Figure 5 for the first method using resistive heating indicate that our microscale platform could perform melting-curve analyses on duplex structures, as well as ligand-based stabilization studies. Stabilization effects (higher Δ*T_m_*) were observed for proflavine, which intercalates between the base pairs [37,38], and DMZ, which binds to the minor groove of duplex DNA [39,40]. An example of a SWV acquired during the melting of the duplex DNA is shown in Figure 5a. A five-point moving average was performed to smooth the electronic noise. Although a platinum pseudo-reference was used, the potential of MB was highly consistent, and the potential did not drift significantly over several experiments. 

To start an experiment below room temperature (20 °C) using resistive heating, an icepack was placed in a polystyrene foam cooler and the entire device platform was positioned inside. Although the same icepack and insulating box were used day-to-day, small variations in the temperature and thus the thermal load on the microheater could occur. The resulting heating profile can vary slightly to produce slightly increased standard deviations (Table 1). In addition, over time there can be some loss of integrity of the device’s insulating oxide layer, which leads to the observation of leakage current (noise) in the SWV that can become problematic in typically less than 10–12 melting profile analyses. The resistive heating data collected over three devices produced similar results. 

We also investigated data collection with the second method of cooling/heating using a thermoelectric module. Though inherently less integrated, this approach offers the convenience of being able to vary the current direction of the thermoelectric to both cool and heat the samples when placed on the backside of the electrochemical platform, removing the need for an ice box. The PRT can still be used (even with devices having a degraded insulating layer due to chemical and thermal stress from multiple experimental cycles) to record the temperature in thermoelectric-driven experiments as long as the monitoring voltage is not applied while the electrochemistry is running. This extends the lifetime of the devices (typically over 30 thermal profiles), while the PRT still provides proximal temperature measurements. Figure 6 shows the data collected with cooling and heating with a thermoelectric module and monitoring the temperature with the PRT. 

Since Δ*T_m_*, _DNA_ was approximately zero, −0.3 ± 0.4 °C (all uncertainties reported as the standard deviation of at least three replicates unless otherwise noted), the duplex DNA melting profile is highly reversible, and the successive measurements do not affect the thermal stability of the duplex. This can be clearly seen in the two sequential melting profiles in Figure 5b and Figure 6b. This sequential procedure may not be appropriate for more complicated biomolecular systems, but it was used in this foundational study to more thoroughly probe the capabilities of the platform and decrease measurement variability. As expected, the *T_m_* values measured for immobilized DNA are lower than the corresponding solution-phase measurements [23,33,34]. For example, the solution-phase *T_m_* derived for 2 µmol/L DNA with 100 mmol/L NaCl would be approximately 32 °C. The example *T_m_* was calculated using OligoAnalyzer* provided by Integrated DNA Technologies (www.idtdna.com/calc/analyzer). Concerns with doing sequential melts involve uncertainty of the stability of the 6-mercaptohexanol SAM, and whether any desorption of DNA from the surface occurs [23]. With our platform and microfabricated electrodes, however, no changes in the baseline response were observed after melting the DNA duplexes at least three times with temperature excursions up to 60 °C. 

The shift in the melting temperatures caused by the two different ligands are seen in Figure 5c,d and Figure 6c,d). The stabilization shifts (all positive) are summarized for sets of triplicate measurements (unless otherwise noted) in Table 1. The initial melting profile of the duplex DNA is shown in black with each set of curves in Figure 5 and Figure 6. Ligand-induced curves were produced after allowing the sample to cool for 15 min and then adding 1 µL of 140 µmol/L ligand in PBS to the PDMS chamber. The sample containing the ligand was allowed an additional 20 min to equilibrate at 10 °C before starting the second melting analysis. Note that these experiments were done on several different devices (Figure S4), as well as on different days.

The results indicate that the electrochemical microplatform can detect and quantify the *T_m_* shift of the duplex DNA arising due to the binding of ligands. Both distinct heating/cooling methods we employed showed the expected positive Δ*T_m_* in the presence of the two ligands. Additionally, the difference in magnitude of Δ*T_m_*_, DMZ_ and Δ*T_m_*, _proflavine_ was approximately 2.5 °C for both methods, which shows self-consistency between the two different set ups. In both experimental methods, the difference in Δ*T_m_* was large enough to discriminate the binding affinities of the ligands, and showed proflavine, a well-known intercalator, had the smaller magnitude shift compared to DMZ, a minor groove binder. 

The automation of the experimental program used in these reported studies has greatly improved measurement repeatability over manually running each SWV and then increasing the voltage to the heater. The limitation of the current automation program is that it supplies a fixed voltage, and as a result, the power applied to heat the well is proportional to 1/*R*^2^. Therefore, when applying a fixed voltage ramp to devices that may have a slight difference in the resistance, the power input and the resulting temperature ramp can show small changes between devices. To reduce this factor, a proportional integral derivative (PID) controller can be included in the automation so that a fixed resistance or temperature set-point could be reached at each interval. 

The motivation of this study was to develop platform capabilities and investigate performance levels as steps toward realizing an automated system for which an array of these microplatforms could be used to simultaneously probe a variety of conditions that alter biomolecular characteristics. Fabrication of a reliable array platform would enable investigations of mechanistic effects and binding kinetics in a highly efficient manner.

## 4. Conclusions

In summary, we used an improved temperature-controlled electrochemical microplatform with the ability to perform rapid melting-curve analyses on duplex DNA in the presence/absence of the binding ligands. By acquiring the peak current values at different temperature values, we were able to construct melting-curves to determine the *T_m_* of different DNA analytes. The obtained Δ*T_m_* values that develop in the presence of the binding ligands show the stabilizing effect these compounds can have on duplex DNA. We demonstrated the measurement of these shifts with two different cooling/heating approaches with good reproducibility observed over several days with different devices. We fully expect that this microscale platform may have more general utility for determining temperature-dependent stability in other types of biomolecular interaction studies. The fabrication methods we used for this microscale device can be extended to produce systems of arrays with incorporated microfluidics, for high-throughput, parallel measurements of binding ligands and other biomolecular system screening, on small-volume samples. 

## Figures and Tables

**Table 1:** Summary of duplex thermal profiles.

	Thermoelectric Cooling/Heating			Refrigerated w/Resistive Heating		
	No ligand	Proflavine	DMZ	No Ligand	Proflavine	DMZ
*T_m,_*_baseline_ (°C)	17.0 ± 1.0	18.5 ± 0.1	16.8 ± 1.0	18.1 ± 1.8	11.8 ± 1.2 ^1^	15.8 ± 0.8
*T_m_*, _control_ (°C)	17.0 ± 1.0	-	-	18.1 ± 2.2	-	-
*T_m_*, _ligand_ (°C)	-	30.3 ± 0.4	31.5 ± 2.0	-	21.9 ± 0.2 ^1^	28.1 ± 1.6
**Δ*T_m_* (°C)**	**−0.3 ± 0.4**	**11.8 ± 0.3**	**14.6 ± 0.6**	**0.0 ± 1.1**	**10.1 ± 1.0 ^1^**	**12.3 ± 1.7**

Standard deviations given for three replicates except for ^1^ (*N* = 2).

## Supplementary Materials

The following are available online at https://www.mdpi.com/2079-6374/9/2/54/s1, Figure S1: Calibration curve of platinum microheater/PRT, Figure S2: Photo of the assembled platform for thermoelectric heating, Figure S3: Full temperature program of platform in a melting experiment, and Figure S4: Duplex melting curves on two different devices on the same day.

## Notes

* Commercial equipment and materials are identified to adequately specify certain procedures. In no case does such identification imply recommendation or endorsement by National Institute of Standards and Technology, nor does it imply the materials or equipment identified are necessarily the best available for the purpose.
