# Supplementary material for: Ligand-Based Stability Changes in Duplex DNA Measured with a Microscale Electrochemical Platform
Source: Biosensors (Basel). 2019 Apr 12;9(2):54. doi: 10.3390/bios9020054 (PMC6628196; doi:10.3390/bios9020054)
Supplement: Supplementary file 1 [file biosensors-09-00054-s001.pdf]

Supplementary Materials:

Ligand-Based Stability Changes in Duplex DNA Measured with a Microscale Electrochemical Platform

Sarah M. Robinson [1,2,3], Zuliang Shen [1,3 †], Jon R. Askim [3], Christopher B. Montgomery [3], Herman O. Sintim [2,*] and Steve Semancik [3,*]

[1] Department of Chemistry and Biochemistry, University of Maryland, College Park, MD 20742, USA; smenio@umd.edu (S.M.R.); zuliangs@gmail.com (Z.S.)
[2] Department of Chemistry, Institute for Drug Discovery, Purdue University, West Lafayette, IN 47907, USA
[3] Biomolecular Measurement Division, National Institute of Standards and Technology, Gaithersburg, MD 20899, USA; jon.r.askim@gmail.com (J.R.A.); chip@nist.gov (C.B.M.)
* Correspondence: hsintim@purdue.edu (H.O.S); stephen.semancik@nist.gov (S.S.)
† Now at Spectrix Analytical Services, LLC, North Haven, CT 06473, USA.

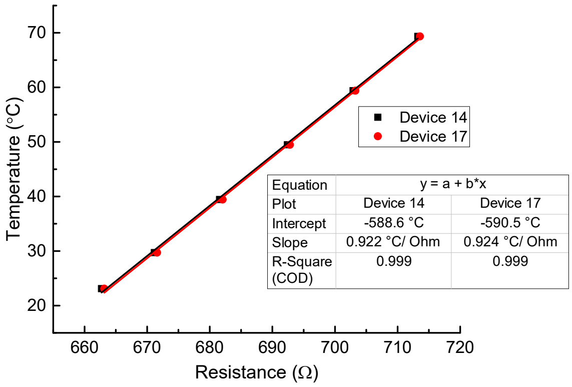

Figure S1. Example calibration curve of platinum microheater/PRT.

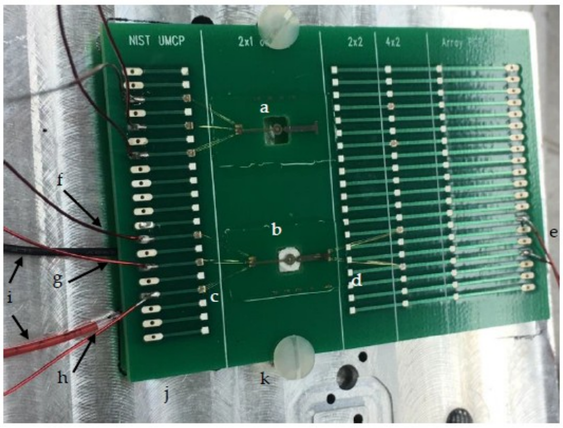

Figure S2. Photo of the assembled platform for thermoelectric heating. (**a**) Device mounted on PCB with epoxy. Platform includes: (**b**) devices mounted on PCB above thermoelectric (TE) module with silicone heat-sink compound at the TE/glass interface, (**c**) wire bonds from contact pads of device electrodes to PCB, (**d**) wire bonds from contact pads of PRT to PCB, (**e**) leads to PRT, (**f**) lead to Au working electrode, (**g**) lead to Pt reference electrode, (**h**) lead to Pt counter electrode, (**i**) leads to TE module (under device), (**j**) Al block heat sink, and (**k**) screws used to secure PCB to Al block. RTV pillars under PCB are not shown.

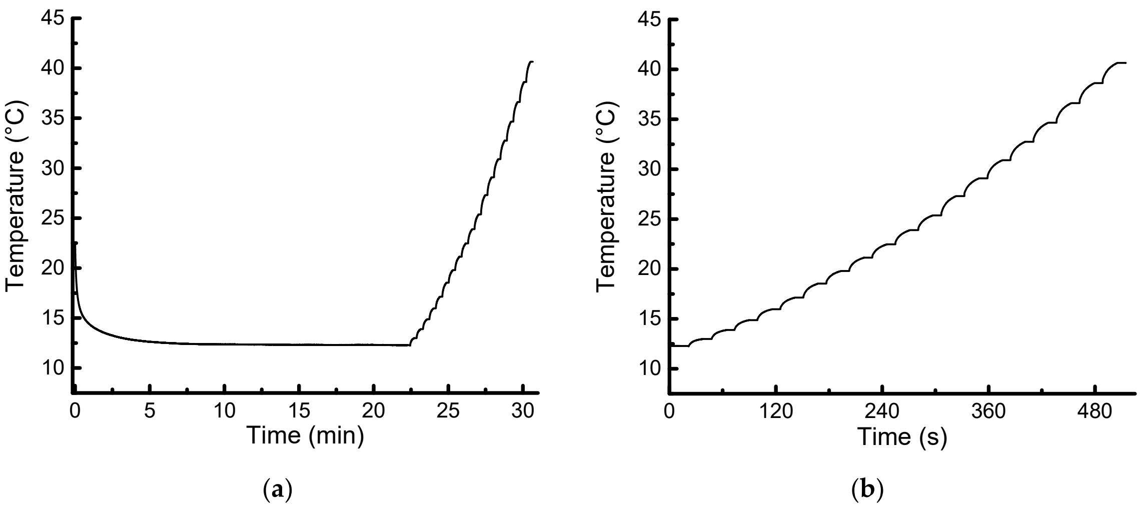

Figure S3. (**a**) Full temperature program of platform in a melting experiment with 20 min of cooling at 0.3 V applied to thermoelectric, and (**b**) an expanded view of the melting portion of the experiment. The voltage was stepped by 0.25 V every 25 s from 0.3 V to −0.3 V.

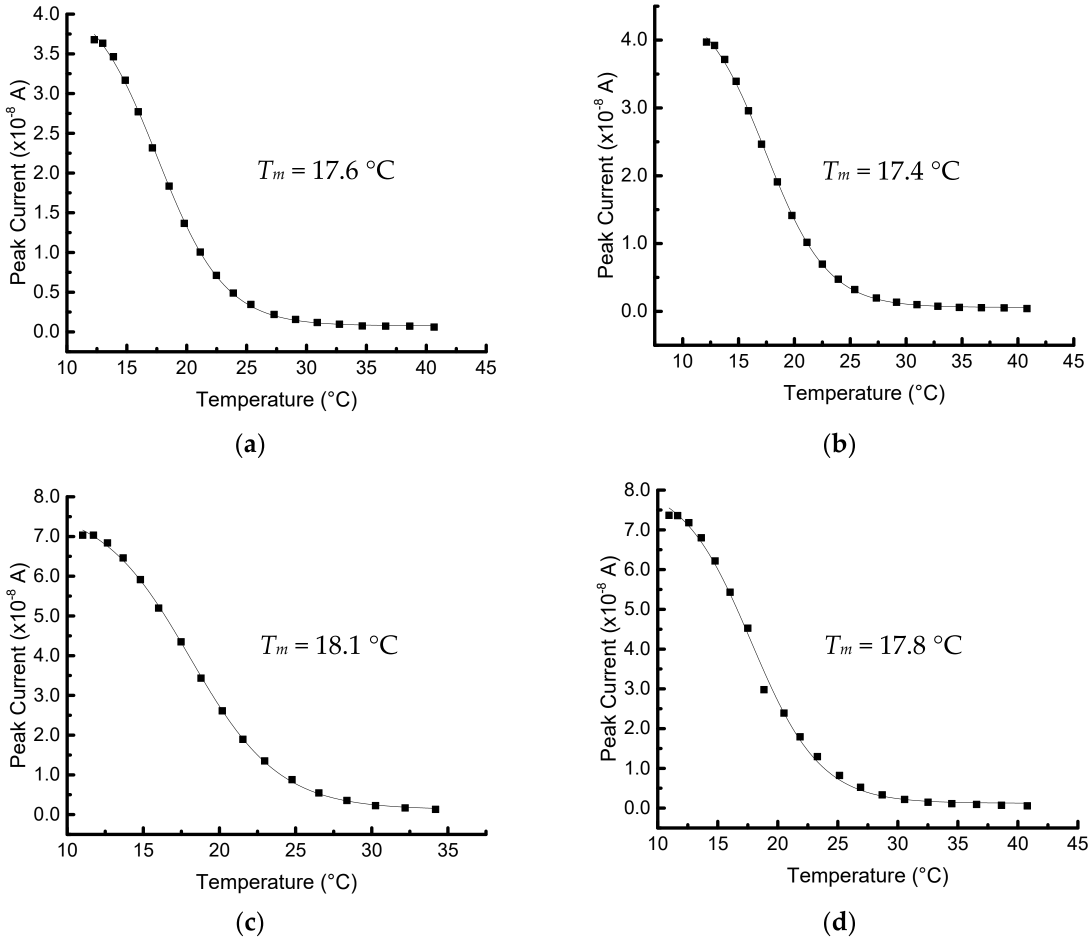

Figure S4. Duplex melting curves on two different devices on the same day. (**a**) and (**b**) show results for Wafer 8, Device 17, trials 1 and 2; (**c**) and (**d**) show results for Wafer 8, Device 14, trials 1 and 2. All data were plotted in OriginPro 2016 and a Boltzmann function was used fit to the data to find T_m.
